# Equivocal Evidence for Colony Level Stress Effects on Bumble Bee Pollination Services

**DOI:** 10.3390/insects11030191

**Published:** 2020-03-18

**Authors:** Arran Greenop, Nevine Mica-Hawkyard, Sarah Walkington, Andrew Wilby, Samantha M Cook, Richard F Pywell, Ben A Woodcock

**Affiliations:** 1 UK Centre for Ecology & Hydrology, Maclean Building, Crowmarsh Gifford, Wallingford, Oxfordshire OX10 8BB, UKrfp@ceh.ac.uk (R.F.P.); bawood@ceh.ac.uk (B.A.W.); 2 Lancaster Environment Centre, Library Avenue, Lancaster University, Lancaster LA1 4YQ, UK; a.wilby@lancaster.ac.uk; 3 Core Research Laboratories, Natural History Museum, Cromwell Rd, Kensington, London SW7 5BD, UK; s.walkington@nhm.ac.uk; 4 Biointeractions and Crop Protection Department, Rothamsted Research, Harpenden, Herts AL5 2JQ, UK; sam.cook@rothamsted.ac.uk

**Keywords:** bumblebees, Neonicotinoid, pollination ecosystem services, pesticide, climate change, heat stress

## Abstract

Climate change poses a threat to global food security with extreme heat events causing drought and direct damage to crop plants. However, by altering behavioural or physiological responses of insects, extreme heat events may also affect pollination services on which many crops are dependent. Such effects may potentially be exacerbated by other environmental stresses, such as exposure to widely used agro-chemicals. To determine whether environmental stressors interact to affect pollination services, we carried out field cage experiments on the buff-tailed bumble bee (*Bombus terrestris*). Using a Bayesian approach, we assessed whether heat stress (colonies maintained at an ambient temperature of 25 °C or 31 °C) and insecticide exposure (5 ng g^-1^ of the neonicotinoid insecticide clothianidin) could induce behavioural changes that affected pollination of faba bean (*Vicia faba*). Only the bumble bee colonies and not the plants were exposed to the environmental stress treatments. Bean plants exposed to heat-stressed bumble bee colonies (31 °C) had a lower proportional pod set compared to colonies maintained at 25 °C. There was also weak evidence that heat stressed colonies caused lower total bean weight. Bee exposure to clothianidin was found to have no clear effect on plant yields, either individually or as part of an interaction. We identified no effect of either colony stressor on bumble bee foraging behaviours. Our results suggest that extreme heat stress at the colony level may impact on pollination services. However, as the effect for other key yield parameters was weaker (e.g. bean yields), our results are not conclusive. Overall, our study highlights the need for further research on how environmental stress affects behavioural interactions in plant-pollinator systems that could impact on crop yields.

## 1. Introduction

Climate change represents a myriad of risks to agricultural production, including the spread of novel pests and diseases as well as direct impacts on yields in response to extreme weather conditions like drought [1,2,3,4]. Climate change may lead to changing complexes of beneficial insects that support key ecosystem services, including the pollination of globally important crops like nuts, fruits and oilseeds [5,6]. Pollination has been suggested to play a role in maintaining yields where crops have been heat stressed, an event likely to be increasingly common in response to changing climatic conditions [7,8]. For instance, it was found that yield losses in bean plants resulting from heat stress could be reduced where plants were pollinated by the bumble bee *Bombus terrestris (Hymenoptera: Apidae)* [7]. However, different species of pollinators show varying tolerances to heat (in terms of their ability to withstand heat stupor) [9]. For example, species with very broad distributions, such as *Bombus lucorum,* have been shown to have a high tolerance to a range of temperatures when compared to more geographically limited species, such as *Bombus flavidus* [9]. While threshold responses to temperatures may directly impact on survival [9,10], there are likely to be a spectrum of responses that result in reduced fitness or changes in behaviour [11,12]. For example, heat stress can act at the level of the individual by affecting their thermoregulatory ability [9]. For social species, complex colony level responses have also been observed [13,14]. High temperatures were shown to reduce the number of foraging bouts undertaken by bumble bees [15], while increasing colony foraging activity in honey bees as a result of a 70% increase of the activation of workers foraging for water [12]. Whilst this demonstrates colony level adaptation, such compensatory behaviours may decrease resilience to other environmental stresses commonly encountered in agricultural situations [12].

Whilst biotic pollination offers the opportunity for yields to be maintained under temperature extremes in some plants [7], this mechanism is highly dependent on pollinators themselves being resilient to other environmental pressures, of which insecticides are one of the most commonly encountered [16,17,18,19,20]. Neonicotinoid insecticides are some of the most commonly used pesticides worldwide. Their systemic use as seed treatments has resulted in residual levels being detected in the pollen and nectar of flowering crops [21]. This has been shown to reduce bee overwintering survival [16], colony growth [22] and pollination services [23]. The impact of neonicotinoids may directly affect behavioural interactions between crops and their pollinators, with evidence suggesting that neonicotinoids can cause a reduction in the frequency of *B. terrestris* foraging bouts [23]. Neonicotinoids also affect the responses of some pollinators to climate, both at the level of the individual [24] and the colony [25]. For example, impaired nest thermoregulation has been found in bumble bees when exposed to imidacloprid [25]. However, the negative effects of neonicotinoids may be dependent on both the compound and the level of exposure seen under field conditions [26,27].

In this study, we examined how changes in the ambient temperature surrounding bumble bee colonies (*B. terrestris* ssp. *audax*) affected both bumble bee behaviour while interacting with flowers and the pollination services that they ultimately provided. We assess this for faba beans (*Vicia faba: Fabaceae*), an important fodder and food crop [7,28]. We also considered the effects of heat stress as it interacts with clothianidin, a widespread neonicotinoid insecticide in global use with established sub-lethal effects on bees [16,29,30]. We test the predictions: 1) Heat stress will cause behavioural changes within colonies that will negatively impact on plant yields as a result of reduced foraging rates, resulting from compensatory behaviours in the colony such as increased nest fanning in an attempt to cool brood chambers [13,15]; 2) the magnitude of these effects will be exacerbated by sub-lethal exposure to the neonicotinoid clothianidin, a class of insecticides known to both affect nest thermoregulation in *B. terrestris* [25] and reduce crop visitation rates [23].

## 2. Materials and Methods 

### 2.1. Experimental Set up

We exposed commercially available colonies of *B. terrestris* to four treatments defined by a 2 × 2 factorial design of: (1) no heat stress and no insecticide exposure (control) (I−H−); (2) heat stress only (I−H+); (3) insecticide exposure only (I+H−); and (4) insecticide exposure and heat stress (I+H+). The details of these treatments are given below. To undertake these experiments, 20 *B. terrestris* colonies were sourced (Biobest, Belgium) on three occasions (eight colonies in June 2018; eight colonies in July 2018; and four in August 2018). Each colony was a 2-week old ‘early colony’ and contained between 26 to 60 workers (mean = 42 SE= 1.80). There was no significant difference in the number of individuals per colony between treatments at the start of the experiment (negative binomial GLM: Χ^2^_3_ = 1.19; *p* = 0.76). Each colony was weighed before being deployed in the experimental treatments. All research was carried out at the Centre for Ecology and Hydrology, Wallingford, UK (54.0093° N, 2.7862° W).

#### 2.1.1. Insecticide Stress 

Each bumble bee colony was housed in a hive with a transparent lid into which two feeding syringes were inserted, allowing bees to feed freely on a 40% sucrose solution used as an artificial nectar source (See Supplementary Material S1; Figure S1). The sucrose solution was either untreated (I−H− and I−H+ treatments) or contained 5 ng g^-1^ *w*/*w* clothiandin (I+H− and I+H+ treatments) (Sigma Aldrich). We used 5 ng g-1 *w*/*w* clothiandin as this is within the middle of the range of field realistic doses that bees could be exposed to in agricultural ecosystems [15]. Colonies were fed on the sucrose solution ad libitum and were provided with honeybee-collected pollen presented in a dish (height = 30mm, diameter = 35mm) (Biobest, Belgium) as a protein source. Colonies were kept indoors in a dark controlled environment room (23 °C) for an initial insecticide exposure phase. The length of the indoor period varied between blocks, depending on when the colony arrived and suitable outside weather conditions for the experiment (indoor duration Block 1 (June) = 9 days; Block 2 (July) = 10 days and Block 3 (August) = 13 days). Colonies were then moved outdoors into cages (see below) and fed on their assigned sucrose treatment for the duration of the experiment (total exposure time Block 1 = 15 days (eight colonies); Block 2 = 15 days (eight colonies), and Block 3 = 18 days (four colonies)). The total duration of insecticide exposure therefore differed between experimental blocks, however, all exposures fall within the range of oilseed rape flowering periods [31], a crop which is a common source of exposure to clothianidin for bumble bees [16]. The only time colonies did not have access to their assigned sucrose solution was during experimental observations on plants when inserted into the cages (see below).

#### 2.1.2. Field Cages

Following the indoor period, colonies were moved to outdoor cages (L 2.5 m × W 1.35 m × H 1.25 m; 4 mm mesh) to acclimatize the bumble bees to outdoor conditions and provide an opportunity for them to learn to forage outside the colony boxes. During this period, each colony was kept in an insulated polystyrene box (L 400 mm × W 300 mm × H 260 mm) with an opening at one end allowing them to enter and exit for the purpose of foraging (Supplementary Material S1; Figure S2a). Feeding syringes were removed from colony boxes and hung at the end of the enclosure to further encourage bumble bees to forage outside the hive [23,32]. The sucrose solution in the syringes appropriate to each insecticide treatment was replaced daily and colonies were fed on this ad libitum with similar access to pollen. Bumble bees were allowed a 48 h foraging period on the feeders at the end of the cage before the heat stress treatments (described below) were applied.

#### 2.1.3. Heat Stress

A heat mat (279.4 mm × 279.4 mm Habistat 25 watt heat mat, Hayes, London, UK) was inserted at the top of each polystyrene box housing the bumble bee colonies. This was attached to a thermostat (Inkbird ITC-308 Digital Temperature Controller, Shenzhen, China) (Supplementary Material S1; Figure S2b) and was used to manipulate the temperature inside the polystyrene container. Heat stress treatments involved raising the ambient temperature of the colony box to either: (1) 25 °C (actual level: mean = 25.25 °C ± 1SD 1.76 °C) for I−H− and I+H− treatments, or (2) 31 °C (actual level: mean = 31.71 °C ± 1SD 1.34 °C) for I−H+ and I+H+ treatments. In both cases, heat was applied between the hours of 10:30 to 16:30 to coincide with the hottest part of the day and peak activity levels of *B. terrestris* [33]. The heat stress was only applied to the colonies and as such the field cage and test crop plants were exposed to common background environmental conditions. The base line target control temperature of 25 °C was chosen because temperatures between 25–30 °C have been found to lead to less than 20% of the colony fanning in *B. terrestris* [13,34], whereas when temperatures exceed 30 °C, bumble bees have been shown to switch from mainly brood maintenance to fanning behaviour (with up to 60% of the colony carrying out fanning behaviour) [13,34]. Our use of short-period high-temperature treatments mimic episodic extreme heat events predicted to become more frequent under a 1.5 °C rise in global temperatures [35]. Availability of crop plants in anthesis meant the time over which the heat stress treatments were applied, while standardised within block, varied between them (Duration of stress: Block 1 = four days, Block 2 = three days, Block 3 = three days). We did not measure ambient temperatures inside the field cages, but do account for diurnal variation in external conditions in the statistics using a Day random effect (See statistical analysis).

#### 2.1.4. Crop Pollination and Foraging Behaviour 

Faba bean (*Vicia fabia*—variety ‘The Sutton’) were grown from seed in 13 L pots (one plant per pot) in a controlled environment greenhouse (16:8 light/dark; 18 °C:15 °C day/night). Multiple cohorts were grown from April–August 2018. This species of broad bean is most often harvested fresh and is an extreme dwarf variety, compared to the field bean variety often used in agriculture but due to its size was selected for practical reasons and has the same flower structure as varieties commercially grown. We marked and numbered between five and six individual clusters of flowers on a plant using colored cable ties [23]. The number of clusters of flowers depended on the number of flowers in anthesis at the start of the experiment. Each cluster consisted of between two and four flowers in anthesis. All other flowers not included in a cluster were removed to standardize the number of flowers available to bees between treatments and replicates. There was no significant difference in the number of flowers between treatments (poisson GLM: *Χ^2^_4_* = 0.64, *p* = 0.96) or clusters between treatments (quasibinomial GLM: *Χ^2^_4_* = 2.51, *p* = 0.64). Following methods outlined in [23], we carried out pilot observations to determine the amount of time bumble bees had access to the plants without causing over-pollination or damage to the plant. Based on these observations, a single plant was placed in a cage with a bumble bee colony for either 25 min (where five flower clusters were available) or 30 min (where six flower clusters were available). A new plant was used for every replicate.

#### 2.1.5. Foraging Behaviour

While bumble bees were foraging on plants, we quantified key aspects of foraging behaviour (Table 1). We observed a single cluster of flowers for 5 min. During this time, we recorded the total number of visits by bees to all flowers on that cluster and for each visit whether the bee legitimately foraged (characterized by the bee entering the front of the flower), nectar robbed (where an individual bites a hole at the base of the flower and consumes nectar) or failed to actively forage on the flower (where a bee does lands on a flower but does not enter the front of the flower to forage or nectar rob). In sequential order, this process was repeated for each of the flower clusters present on the plant. Colonies were individually randomly sampled between 10:30 am and 4:30 pm during the application of the heat treatments following the above process. Each colony was sampled on either 2 or 3 separate days, where possible with at least one colony from each treatment sampled each day (except where colonies were removed due to weather or the number of flowers in anthesis limited this (in Block three; Day 3). In all cases, we paired each plant exposed to bumble bees with a separate caged control (with no bumble bees).

### 2.2. Assessing Pollination Effects on Seed Set

After the plants had been exposed to the bumble bees, they (and the controlled plants, exposed for the same duration in outside cages without bees) were returned to the controlled environment greenhouse so that they could mature and set seed pods. Once ripe (R7 growth stage: pod formation), seeds were harvested and then oven dried [40]. Total number of whole pods were counted, as well as the number and total weight of de-husked beans within them.

### 2.3. Statistical Analysis 

#### 2.3.1. Behaviour

We wanted to determine whether the colony stress treatments affected bumblebee foraging behaviours that could in turn affect crop yields. We tested for differences between treatments for legitimate visits per 5 min, number of non-foraging visits per 5 min, and probability of nectar robbing. We used Bayesian generalised mixed models (BGLMM) implemented in the brms package to determine the effect of colony stress on bumble bee behaviours using RStudio [41,42,43]. For the legitimate and non-forage visitation response variables, we used a negative binomial distribution with log link function (to account for overdisperison) and for nectar robbing a Bernoulli BGLMM with logit link function. The negative binomial models were run with a vague Normal (mean = 0, standard deviation = 100) prior and the Bernoulli model with a Normal (0, 2.5) prior on the intercept and fixed effects, which places a low mass on extreme values on the probability scale [44]. A half student *t* prior with 3 degrees of freedom was placed on the random effects, which is the default in brms [42]. We first tested whether there was any support for the interaction between Heat and Insecticide by running a model with the main effects Heat (H- and H+) and Insecticide (I- and I+) + random effects, and another model containing the interaction between Heat × Insecticide + random effects. All models included the random effect Plant ID nested in Colony ID crossed with Day. Colony ID was included to account for the fact that multiple plants were pollinated by the same colonies, Day for differences in environmental conditions that could also impact on colonies any given day between temporal Blocks [45] and Plant ID as multiple observations came from a single plant. The main effects and interaction models were compared using k-fold (k = 10) cross validation, which estimates the predictive error of a model [46]. We selected the model with the lowest prediction error, or where there was no significant difference (value of the difference is at least five times that of the standard error [47]) between models we chose the simplest model based on parsimony. We used four chains each run for 4000 iterations with 1000 burn in iterations. Model fit was assessed based on *Rhat* values (<1.05) to ensure chain convergence and by carrying out posterior predictive checks and inspection of residual plots [48]. We calculated the mean posterior distribution of differences between treatment levels and 95% credible interval (CI). Where CI for treatment differences did not overlap zero, we concluded that there was evidence of an effect of that parameter. The queen of one of the colonies assigned to I+H+ (Block 1 in June) treatment died within 7 days of colony arrival, thus observations were removed for this colony, which reduced the sample size to four for this treatment. Also, abnormally hot weather in the UK during July (Block 2) meant that four colonies had to be removed belonging to the I−H− and I+H− as their temperature could not be maintained below 29 °C, therefore, confounding the low and high temperature treatments (number of colonies and plants per treatment after exclusions: I−H− = 3 colonies, 8 plants; I+H− = 3 colonies, 9 plants, I−H+ = 5 colonies, 13 plants; and I+H+ = 4 colonies, 11 plants).

#### 2.3.2. Plant Yields

We wanted to determine whether the colony stress treatments affected the pollination effectiveness of bumble bees. We firstly investigated whether exposure to bumble bees affected yields compared to the control plants that were not exposed to bumble bees. This was done to ensure any effects of the colony stress treatments on plant yields were not due to fluctuations in bean yield independent of bumble bee exposure. Because 70.73% of control plants failed to produce pods, this led to zero inflation, thus we determined if bumble bee exposure increased the probability of a plant producing pods using a Bernoulli distributed response variable (Table 2) [49]. We first included bumble bee exposure as a five-level factorial explanatory variable (Control, H-I-, H-I+, H+I- and H+I+), however, this led to partial separation due to the fact that there was no variation in the response of I+H− replicate plants (only one value in the response), leading to inflated parameter estimates and standard errors [50]. Consequently, we grouped all plants exposed to bumble bees (n = 41) and compared them to the control plants, which shows whether pollination overall had an effect on the probability of plant producing any pods at all (n = 41). Within this statistical model bumble exposure (control and bee exposed) was set as the fixed effect, with the random effects of Colony ID crossed with Day. Exposure to bumble bees increased the probability of a plant producing pods (control log odds = −1.27 [lower CI = −2.84, upper CI = −0.19]); difference in bumble bee exposure log odds = 2.86 [lower CI = 1.34, upper CI = 5.18]). Therefore, we then carried out separate models focusing only on plants that had been exposed to bumble bees where the data was not zero inflated to determine the effects of the colony stress treatments on pollination services for the yield variables listed in Table 2 (additional yield variables are included in Supplementary Material S2). We again tested support for the interactions by running a model with the main effects Heat (H- and H+) and Insecticide (I- and I+) + random effects, and another model containing the interaction between Heat × Insecticide + random effects using k-fold selection. All models contained the random effects Colony ID crossed with Day unless otherwise specified in Table 2. We used four chains each run for 4000 iterations with 1000 burn-in iterations. Models were validated using the same protocol as for the behavioural variables.

## 3. Results

### 3.1. Behaviour Responses to Heat Stress and Pesticide

A total of 1489 interactions between plants and bees were observed over the experimental period. Of these interactions, 70.65% were legitimate foraging events, where the bee entered the front of the flower, 26.59% were non-foraging visits and 2.75% were nectar robbing visits. We found no support that the interaction Heat × Insecticide increased the predictive accuracy of any of the behavioural variable models (Table 3). The main effects Heat and Insecticide also showed no clear effect on the number of legitimate visits, non-foraging visits and the probability that a plant would be nectar robbed (Table 4). 

### 3.2. Yield parameters 

**Figure 1:**
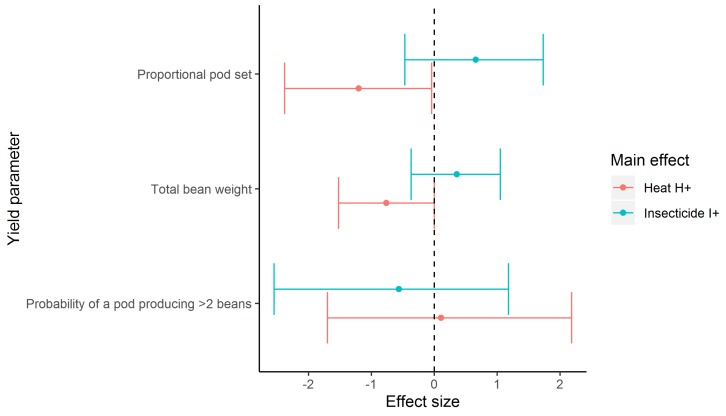
The effect size for the main effects Heat stress H+ (colonies raised to 31 °C) and Insecticide I+ (colonies reared on sucrose solution containing (5 ng g^-1^ *w*/*w* of clothianidin insecticide), which represents the difference from H- (colonies maintained at 25 °C) and I- (colonies raised on just sucrose solution) for each yield parameter. Error bars are 95% credible intervals. Where error bars do not overlap zero is evidence of an effect on the yield parameter.

When we analysed just the colony stress treatments, we found no evidence that including the interaction effect in any of our models increased the predictive accuracy (Table 5). However, we found evidence that plants foraged on by bumble bee colonies that were exposed to heat stress (31 °C) had a lower proportional pod set (log odds = −1.20 [lower CI = −2.38, upper CI = −0.04) than those in the 25 °C treatment (subsequently there was also lower total pod weight, see Supplementary Material S2). There was evidence for an effect on bean yields (−0.76 [lower CI = −1.52, upper CI = 0.00]), although the credible interval overlapped zero (Table 6; Figure 1). There was no evidence of a heat effect on the probability that a plant would produce more than two beans per pod (0.11 [lower CI = −1.70, upper CI = 2.18]). Additionally, we found no evidence that colony heat stress impacted on individual pod weight or individual bean weight (Supplementary Material S2). There was no clear effect of insecticide exposure on any of the yield parameters; most yield variables showed a positive effect of insecticide, but the 95% credible intervals all overlapped with zero (Table 6: Figure 1). The raw means and standard errors for each treatment are included in Supplementary Material S2.

## 4. Discussion

### 4.1. Heat Stress

We found equivocal evidence in support of our prediction that heat stress would cause lower plant yields. Where we looked at the treatment effects on yields, our results suggest a negative effect of heat stress on pollination services impacting the pod set, however, an overlap with zero was seen for total bean weight, which is a key yield parameter in faba bean [51,52]. We also found no evidence that yield differences were linked to any changes in the types of foraging behaviour we observed (i.e. legitimate visitation rate or prevalence of nectar robbing).

Faba bean is pollinated via the mechanisms of self- and cross-pollination [52,53]. Pollinators can facilitate both types of pollination as they carry pollen from other plants, which increases cross-pollination, but they also “trip” a physical barrier between the stigma and the anthers that improves self-pollination [54]. As only a single plant was included in each of the experimental cages, it is unlikely our findings on pod set and pod weight relate to behavioural changes in bee foraging that would have impacted on, or limited, cross-pollination. It is possible that differences in temperatures may have caused changes in colony resource demands, due to variations in colony energy expenditure [55,56]. For the species *Bombus impatiens,* it has been shown that at ambient colony temperatures of 32 °C, oxygen consumption was at its minimum and deviations either side of this caused an increase in energy expenditure by colonies [34]. Colony energy expenditure can drive changes in bee foraging behaviour, for example individuals switch from pollen to nectar collection and vice versa to account for whichever resource is in most demand [55,56].

It is difficult to speculate what the impact of heat on pollination behaviour was, as none of the foraging behaviours we observed were affected. Whole suites of morphological and behavioural traits have been found to be correlated with pollination success [57], although a number of studies have successfully used visitation rates as a proxy for pollination delivery [23,37,58], which our study focused on. In the context of our experiment, if the heat stress imposed an energy cost on colonies, then it could be expected that bees would be more likely to collect nectar than pollen as it offers the highest energy reward. Bumble bees may have also been more likely to forage for nectar if the heat treatment imposed water stress. Either way, it could then be expected that heat-stressed colonies would be more likely to nectar rob as this has been demonstrated to be one of the most efficient ways to gain nectar [59]. However, this was not found and nectar robbing overall occurred infrequently in our study. Nectar robbing within *B. terrestris* on faba bean is predominantly driven by whether individuals have been exposed to previously robbed flowers and through social transmission [60]; as individuals in our experiment only foraged on plants for relatively short periods, the time frame for this behaviour to come prevalent was reduced. It is more likely that heat stress caused behavioural changes in the bees’ interactions with the flower that were beyond the resolution with which we observed behaviours. For example, behaviours that may have impacted the plant styles or stigma contact [61,62]. It also cannot be ruled out that as our sample sizes were relatively low and large variation existed between colonies that we had limited power to difficult to detect behavioral effects, particularly at the level of interaction level [63,64].

An important point to highlight is that bean plants are likely to be more vulnerable to direct heat damage than bumble bees [7,9]. For example, at temperatures of 34 °C, pollination was found to have no effect on yield recovery in faba bean, as female organs in plants became damaged and fertilisation was no longer possible [7]. The potential impacts of climate on behavioural interactions between pollinators and plants remains understudied and the extent to which either plants or pollinators are the weak link in systems is difficult to ascertain. It seems likely that plants will often have lower thermotolerances [7,65,66] than their pollinator species [9,11,12], although this may not be the case in all systems [10]. High ambient air temperatures are likely to predominantly affect commercial *B. terrestris* colonies, honeybees and other above-ground nesting bees. As soil temperatures generally remain more stable than surrounding ambient air temperatures [13], belowground nesting species such as wild *B. terrestris* usually have a buffer between high ambient air temperatures to help maintain stable brood thermoregulation [13]. Our method is novel in that it isolates colony level drivers from ambient air temperature effects on plants. However, it also highlights the question of whether there could be additive impacts of high temperatures on pollination ecosystem services and the additional damage this could cause to plants.

### 4.2. Clothianidin Exposure

Exposure to clothianidin insecticide was found to have no effect on *B. terrestris* foraging behaviour and ultimately was found to have no clear effect on yields. In a field study using 5 ng g^-1^ of clothianidin, only subtle effects of exposure on *B. terrestris* foraging behaviours were found over a 5-week period [15]. For example, initially clothianidin exposure increased the proportion of foragers collecting pollen early in the experiment compared to the control, but that these differences disappeared mid-way through the 5-week period [15]. It is difficult to ascertain whether generalisations can be made about whether clothianidin affects pollinators less than other neonicotinoids. While there is evidence to suggest at least at 5 ng g^-1^, it has minimal effects on pollinator behaviour, the manifestation of sub-lethal effects, at least in cage studies, are strongly dose dependent with large variation between experiments [67]. Additionally, our low sample sizes may have impacted on our ability to detect an effect particularly between temporal blocks, which may have led to further variation. While there is variable evidence of the impacts of neonicotinoids on pollination in cage studies, larger scale field studies that look at the natural chronic exposure to neonicotinoids have shown negative impacts on a number of colony and individual characteristics both in domestic and wild pollinator populations [16,22,68]. It is important that cage studies attempt to address the mechanisms driving the effects found in larger field trials and adopt experimental designs that help reconcile the findings between the two approaches to testing the effects of insecticide on pollinators [69].

Many studies, as is the case in the current research, focus on a nominal dose of insecticide when studying sub-lethal effects on behaviours. Often this is done for pragmatic reasons, however, this has limitations as it exposes individuals to a single dose with no choice in food sources, which may lead to artificially high levels of insecticide consumption [15,70]. Adoption of experiments that utilise semi-choice designs where colonies have access to both contaminated food sources and other forage material may prove useful in accounting for foraging preferences and help bridge the gap between mechanistic experiments and larger field studies [15,69]. Another issue with cage experiments, particularly those carried out in the lab, is that they often raise colonies under optimum conditions and focus on single stressors [15]. Our experimental design attempted to address some of these issues utilising a semi-field design and investigating the joint effects of another stressor in addition to insecticide. Even the inclusion of additional stressors does not replicate the level of complexity of field studies and the myriad of environmental drivers that pollinators are exposed to [71]. However, approaches such as ours and those that investigate chemical mixtures [72,73] may prove more useful than considering stressors in isolation. Finally, our study highlights that solely assessing behaviours, such as visitation rates, may not offer the scale required to detect subtle changes in foraging behaviours that could impact on crop yields. For example, those that occur within flowers and are therefore not directly observable during foraging events. To address this, future studies could utilise a design that includes both colony level responses, such as the number of active foragers, and look at fine-scale individual measurements i.e. resource acquisition. This approach has been used to unpick whether individual alterations in a forager’s behaviour or colony adaptations effecting worker-bee numbers impact on yields [23].

## 5. Conclusions

Climate change poses a number of threats to biotic processes [1,74,75,76]. Obvious threats to plant –pollinator systems are perturbations in pollination services delivery due to loss of pollinator species and lower overall abundances across taxa due to changing distributions [71,77,78]. Our study indicates that there may be more subtle effects related to heat stress at the colony level, which in turn could impact on the delivery of pollination services. Methodological issues mean our results are far from conclusive, likely as a result we found variation in effects between the yield variables analysed. In addition, there was no obvious mechanism for why lower yields occurred in terms of the behavioural interactions seen between pollinators and flowers. Consequently, further research is required to determine the extent of these effects, both within the study system used here as well as in other plant-pollinator systems. The synergistic effects of climate change on the various components of crop production could have significant consequences on future food security. Our study, in line with others [74,79,80,81], highlights the importance of investigating how biotic interactions may be affected by climate change and how this in turn could affect ecosystem services upon which humans are reliant.

## Figures and Tables

**Table 1:** Behavioural variables observed to determine the effects of stress on *Bombus terrestris* foraging behaviour on *Vicia faba*. Colony stress treatments were Heat (25 °C or 31 °C) and Insecticide (40% sucrose solution or 40% sucrose solution + 5 ng g-1 *w*/*w* of clothianidin insecticide). Each level of heat treatment was crossed with each level of Insecticide.

Response Variable	Description	Reason for Inclusion
Legitimate pollination visitation rate (number of visits per 5 min interval)	Legitimate visits were classed when a bee entered the front of the flower to forage; this behaviour is most likely to lead to pollination [36].	Often used measure of bee pollination services [23,37,38].
Non-forage visitation rate (number of visits per 5 min interval)	This is the total number of visits to a plant where a bee did not actively forage.	Provides a measure of the level of activity carried out that does not provide any nutritional benefit to an individual or the colony.
Nectar rob (0 = no robbing, 1 = robbed)	Number of nectar robbing visits to a flower cluster. This was modelled as a binary response variable as the nectar robbing occurred in only 2.75% of visits and show little variation across the response.	Nectar robbing is unlikely to have a beneficial effect on pollination [36,39].

**Table 2:** Faba bean (*Vicia faba*) yield parameters and the model structure used to determine the effects of Heat and Insecticide (clothianidin) stress on *Bombus terrestris* pollination services. Control plants were not exposed to bumble bees. Colony stress treatments were Heat (25 °C or 31 °C) and Insecticide (40% sucrose solution or 40% sucrose solution including 5 ng g^-1^ *w*/*w* of clothianidin). Each level of Heat treatment was crossed with each level of Insecticide for the colony stress treatment models only. Priors are expressed as Normal (μ, σ).

**Control vs. Bumble bee exposed plants**	
**Response variable**	**Model description**
Probability of a plant producing pods (binary 0 = no pods produced and 1 = >0 pods produced)	Priors: Weakly informative Normal (0, 2.5) on intercept and fixed effect coefficients, this prior still allows extreme values but places a lower mass on them on the probability scale [44]. Default prior in brms for the random effects. Distribution: Bernoulli with logit link function As 70.73% of control plants produced no pods this was a binary response variable.
**Colony stress treatment models**	
**Response variable**	**Model description**
Proportional pod set per plant (n= 41)	Priors: Normal (0, 2.5) on intercept and fixed effect coefficients [44]. Default prior in brms for the random effects. Distribution: Binomial with logit link function. The proportion of flowers that turned into pods.
Total bean weight per plant (g) (n = 41)	Priors: Normal (0,100) on intercept and fixed effects. Default prior in brms for the random effects. Distribution: Normal. Log+1 transformed. The total weight of all beans per plant.
Number of beans per pods (binary 0 = 2 beans or less or 1 = > 2 beans per pod) (n = 124)	Priors: Normal (0, 2.5) on intercept and fixed effect coefficients [44]. Default prior in brms for the random effects. Distribution: Bernoulli with logit link. This variable was the number of beans produced per pod and followed a uniform distribution across the values 1–4 and was poorly modelled by a poisson or negative binomial response distribution. Included Plant ID random effect.

**Table 3:** The difference in predictive accuracy between the Main effects model (Heat and Insecticide + random effects) and the model including the interaction (Heat × Insecticide +random effects) on *Bombus terrestris* foraging behaviours. The model with the highest predictive accuracy is ranked as 0 with values showing the difference in validation error and standard error of the difference between models.

Behavioural Variable	Model	Difference in Validation Error	Standard Error of the Difference
Legitimate visitation rate	Interaction effects model Main effects model	0 −3.52	0 5.1
Non-foraging visitation rate	Main effects model Interaction effects model	0 −1.38	0 2.17
Probability of nectar robbing	Interaction effects model Main effects model	0 −4.31	0 2.94

**Table 4:** Parameter estimates for the main effects Heat (H- and H+) and Insecticide (I- and I+) on the behavioural variables analysed using Bayesian mixed models. The intercept represents the mean value at the H- (colonies maintained at 25 °C) and I- (colonies reared on surcrose solution), and H+ (colonies maintained at 31 °C) and I+ (colonies reared on sucrose containing 5 ng g^-1^ *w*/*w* of clothianidin) represent the difference between the intercept and these factor levels. Cases where the 95% credible interval show no overlap with zero is evidence for an effect of that parameter on pollinator behaviour.

Behavioural Variables	Parameter	Estimate	Lower 95%CI	Upper 95%CI
Legitimate visitation rate (visits/5 min period)	Intercept Heat H+ Insecticide I+	0.31 0.37 0.03	−1.56 −0.95 −1.21	2.09 1.64 1.24
Non-foraging visitation rate (visits/5 min period)	Intercept Heat H+ Insecticide I+	−0.85 0.64 0.30	−2.17 −0.15 −0.34	0.36 1.46 0.94
Probability of nectar robbing (0 = not robbed and 1 = robbed)	Intercept Heat H+ Insecticide I+	−3.36 −1.79 1.44	−6.15 −4.36 −0.82	−0.86 0.53 3.98

**Table 5:** The difference in predictive accuracy between the Main effects model (Heat and Insecticide + random effects) and the model including the interaction (Heat × Insecticide +random effects) on yield parameters. The model with the highest predictive accuracy is ranked as 0 with values showing the difference in validation error and standard error of the difference between models.

Yield Variable	Model	Difference in Validation Error	Standard Error of the Difference
Proportional pod set	Main effects model	0	0
	Interaction effects model	−5.28	1.66
Total bean weight	Main effects model	0	0
	Interaction effects model	−3.69	1.78
Probability of a pod producing >2 beans	Interaction effects model Main effects model	0 −0.32	0 2.74

**Table 6:** Parameter estimates for the main effects Heat (H- and H+) and Insecticide (I- and I+) on the yield variables analysed using Bayesian mixed models. The intercept represents the mean value at the H- (colonies maintained at 25 °C) and I- (colonies reared on sucrose solution), and H+ (colonies maintained at 31 °C) and I+ (colonies reared on sucrose containing 5 ng g^-1^ *w*/*w* of clothianidin) represent the difference between the intercept and these factor levels. Where the 95% credible intervals do not overlap zero is evidence for an effect of that parameter on yields.

Yield Variable	Parameter	Estimate	Lower 95%CI	Upper 95%CI
Proportional pod set	Control	−1.69	−2.88	−0.50
	Heat H+	−1.20	−2.38	−0.04
	Insecticide I+	0.66	−0.47	1.73
Total bean weight	Control	1.41	0.66	2.18
	Heat H+	−0.76	−1.52	0.00
	Insecticide I+	0.36	−0.37	1.05
Probability of a pod producing > 2 beans	Control Heat H+ Insecticide I+	−0.19 0.11 −0.56	−2.04 −1.70 −2.55	1.54 2.18 1.18

## Supplementary Materials

The following are available online at https://www.mdpi.com/2075-4450/11/3/191/s1, Figure S1: Colony boxes used in the experiment, Figure S2: Photo of the outdoor field cages and polystyrene containers used to house the colonies, Table S1: Additional faba bean (Vicia faba) yield parameters and the model structure, Table S2: The difference in predictive accuracy between the Main effects model and the model including the interaction on the additional yield parameters, Table S3: Parameter estimates for the main effects Heat (H- and H+) and Insecticide (I- and I+) on the additional yield variables analysed using Bayesian mixed models, Table S4: Raw means and standard error for each of the yield parameters analysed for the colony stress treatments.
