# Supplementary material for: Equivocal Evidence for Colony Level Stress Effects on Bumble Bee Pollination Services
Source: Insects. 2020 Mar 18;11(3):191. doi: 10.3390/insects11030191 (PMC7142647; doi:10.3390/insects11030191)
Supplement: Supplementary file 1 [file insects-11-00191-s001.zip › Supp info/Supplementary material S1.docx]

**Supplementary material** **S1: Experimental set up**


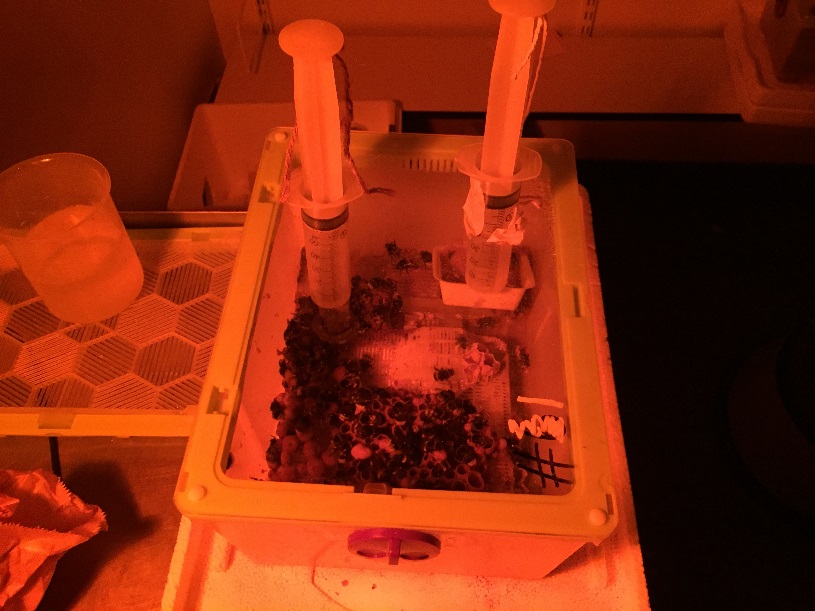


**Figure S1.** Colony boxes used in the experiment. The two feeding syringes are included at the rear of the colony box.


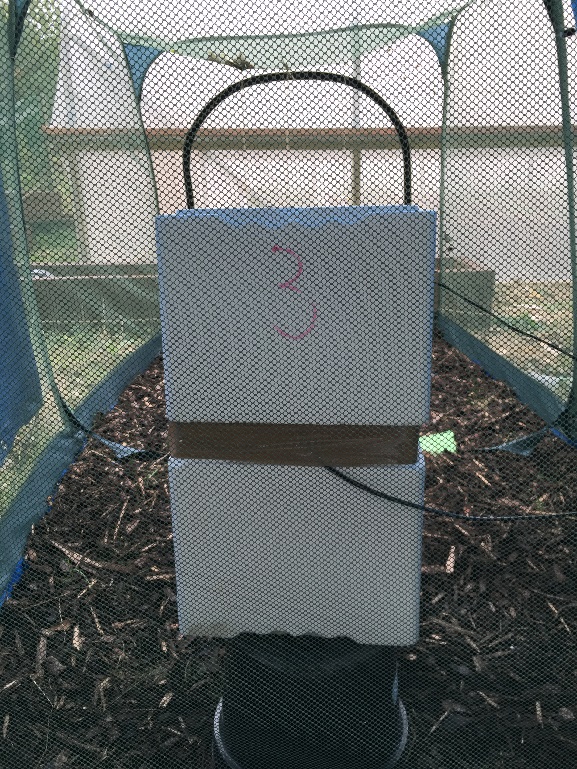


**2b)**

**2a)**

2

1

Hive

Polystyrene container

Heat mat

Temperature probe

Opening

**Figure S2a.** Photo of the outdoor field cages and polystyrene containers used to house the colonies. Number 1 denotes the cable for the heat mat and number 2 the temperature probe. **S2b)** Diagram of the interior of the colony boxes showing the position of the hive and heat mat.
