# Supplementary material for: Equivocal Evidence for Colony Level Stress Effects on Bumble Bee Pollination Services
Source: Insects. 2020 Mar 18;11(3):191. doi: 10.3390/insects11030191 (PMC7142647; doi:10.3390/insects11030191)
Supplement: Supplementary file 1 [file insects-11-00191-s001.zip › Supp info/Supplementary material S2.docx]

**Supplementary material S2: Additional yield parameters and raw means table**

| **Table S1.** Additional faba bean (*Vicia faba*) yield parameters and the model structure used to determine the effects of Heat and Insecticide (clothianidin) stress on Bombus terrestris pollination services. Control plants were not exposed to bumble bees. Colony stress treatments were Heat (25^o^C or 31^o^C) and Insecticide (40% sucrose solution or 40% sucrose solution including 5 ng g^-1^ w/w of clothianidin). Each level of Heat treatment was crossed with each level of Insecticide for the colony stress treatment models only. Priors are expressed as Normal (μ, σ). | |
| --- | --- |
| Total pod weight per plant (g)  (n = 41) | **Priors:** Normal (0,100) on intercept and fixed effects. Default prior in brms for the random effects.  **Distribution:** Normal. Log+1 transformed.  The total weight of all pods per plant. |
| Individual pod weight (g) (n=124) | **Priors:** Normal (0,100) on intercept and fixed effects. Default prior in brms for the random effects.  **Distribution:** Normal. Log transformed.  Included Plant ID random effect. The weight of individual pods. |
| Individual bean weight (g)  (n = 291) | **Priors:** Normal (0,100) on intercept and fixed effects. Default prior in brms for the random effects.  **Distribution:** Normal. Log transformed.  Included Plant ID random effect. The weight of individual beans. |

| **Table S2.** The difference in predictive accuracy between the Main effects model (Heat and Insecticide + random effects) and the model including the interaction (Heat × Insecticide +random effects) on the additional yield parameters. The model with the highest predictive accuracy is ranked as 0 with values showing the difference in validation error and standard error of the difference between models. | | | |
| --- | --- | --- | --- |
| Total pod weight | Interaction effects model | 0 | 0 |
|  | Main effects model | -1.177 | 1.462 |
| Individual pod weight | Main effects model  Interaction effects model | 0  -2.613 | 0  2.989 |
| Individual bean weight | Interaction effects model  Main effects model | 0  -2.531 | 0  4.871 |

| **Table S3**. Parameter estimates for the main effects Heat (H- and H+) and Insecticide (I- and I+) on the additional yield variables analysed using Bayesian mixed models. The intercept represents the mean value at the H- (colonies maintained at 25^oC^) and I- (colonies reared on sucrose solution), and H+ (colonies maintained at 31^oC^) and I+ (colonies reared on sucrose containing 5 ng g-1 w/w of clothianidin) represent the difference between the intercept and these factor levels. Where the 95% credible intervals do not overlap zero is s evidence for an effect of that parameter on yields. | | | | |
| --- | --- | --- | --- | --- |
| Total pod weight | Control | 1.829 | 0.919 | 2.721 |
|  | Heat H+ | -0.912 | -1.778 | -0.049 |
|  | Insecticide I+ | 0.399 | -0.442 | 1.206 |
| Individual pod weight | Control | 0.751 | 0.183 | 1.311 |
|  | Heat H+ | -0.044 | -0.678 | 0.572 |
|  | Insecticide I+ | -0.160 | -0.767 | 0.454 |
| Individual bean weight | Control | -0.615 | -1.338 | 0.079 |
|  | Heat H+ | -0.295 | -1.077 | 0.476 |
|  | Insecticide I+ | -0.061 | -0.812 | 0.687 |

| **Table S4**. Raw means and standard error for each of the yield parameters analysed for the colony stress treatments. Colony stress treatments were Heat (25^o^C or 31^o^C) and Insecticide (40% sucrose solution or 40% sucrose solution + 5 ng g-1 w/w of clothianidin insecticide). Each level of Heat treatment was crossed with each level of Insecticide. Only main effects are shown in the table. | | | | |
| --- | --- | --- | --- | --- |
| **Variable** | **H-** | **H+** | **I-** | **I+** |
| Proportional pod set | 0.27 | 0.12 | 0.13 | 0.22 |
|  | 0.06 | 0.03 | 0.03 | 0.05 |
| Total pod weight per plant (g) | 11.68 | 4.69 | 5.72 | 9.55 |
|  | 2.93 | 1.38 | 1.68 | 2.58 |
| Total bean weight per plant (g) | 6.92 | 2.53 | 3.08 | 5.68 |
|  | 1.9 | 0.73 | 0.91 | 1.66 |
| Number of beans per plant pod | 2.36 | 2.26 | 2.29 | 2.34 |
|  | 0.11 | 0.15 | 0.14 | 0.11 |
| Individual pod weight (g) | 2.54 | 2.45 | 2.67 | 2.42 |
|  | 0.14 | 0.19 | 0.18 | 0.14 |
| Individual bean weight (g) | 0.63 | 0.58 | 0.63 | 0.60 |
|  | 0.02 | 0.03 | 0.03 | 0.02 |
